# Multiscale Segmentation-Guided Diffusion Model for CBCT-to-CT Synthesis

**DOI:** 10.3390/life15121871

**Published:** 2025-12-06

**Authors:** Yike Guo, Yi Luo, Hamed Hooshangnejad, Rui Zhang, Xue Feng, Quan Chen, Wilfred Ngwa, Kai Ding

**Affiliations:** 1 Department of Biomedical Engineering, Johns Hopkins University, Baltimore, MD 21287, USA; 2 Department of Radiation Oncology and Molecular Radiation Sciences, Johns Hopkins University, Baltimore, MD 21287, USA; 3 Division of Computational Health Sciences, Department of Surgery, University of Minnesota, Minneapolis, MN 55455, USA; 4 Department of Biomedical Engineering, University of Virginia, Charlottesville, VA 22908, USA; 5 Department of Radiation Oncology, Mayo Clinic Arizona, Phoenix, AZ 85054, USA

**Keywords:** synthetic CT, CBCT, diffusion model

## Abstract

To improve synthetic CT (sCT) generation from cone-beam CT (CBCT) in radiotherapy, we propose a multiscale segmentation-guided diffusion framework. The proposed model integrates anatomical priors across multiple spatial resolutions through a segmentation mask pyramid and introduces a scale-specific loss function to guide learning at each level. When evaluated on the SynthRAD2023 brain dataset, our model achieves a mean absolute error (MAE) of 61.82 HU, a peak signal-to-noise ratio (PSNR) of 32.05 dB, and a structural similarity index (SSIM) of 0.90, outperforming baseline models. These results suggest that multiscale anatomical guidance can improve the fidelity and anatomical consistency of sCT images, thus facilitating high-quality CBCT-to-CT translation in radiotherapy applications.

## 1. Introduction

Image-guided adaptive radiotherapy (IGART) has been widely adopted due to its ability to improve treatment precision. By using real-time imaging to monitor and adapt to anatomical changes, IGART enables dynamic adjustments to radiotherapy plans throughout the course of therapy [1,2]. To support this paradigm, clinical workflows often rely on several imaging modalities, including computed tomography (CT), cone-beam CT (CBCT), and magnetic resonance imaging (MRI). Among these, CT remains the primary adopted imaging modality for treatment planning since it provides electron density information essential for accurate dose calculation [3]. However, repeated CT scans may increase ionizing radiation exposure and introduce additional complexity into clinical workflows [4]. Therefore, CBCT has emerged as a practical alternative to CT. Although CBCT can be seamlessly integrated into linear accelerators and enables frequent imaging, CBCT images suffer from several drawbacks, including artifacts caused by scatter noise, low soft-tissue contrast, and truncated projections, which restrict their direct use in treatment planning [5,6]. Although another substitute, MRI, is free of ionizing radiation and superior in soft-tissue contrast, it is costly and incompatible with conventional radiotherapy infrastructure [7,8]. These challenges have motivated the development of synthetic CT (sCT) techniques, which generate CT-equivalent images from alternative modalities, most notably CBCT, thus facilitating online adaptive radiotherapy without imposing additional radiation burden on the patient [3,9,10].

In recent years, various deep learning models have been applied to improve the accuracy of sCT images, ranging from generative adversarial networks (GANs) to recent conditional diffusion models [11,12]. In particular, conditional diffusion models [13,14] have exhibited outstanding feasibility compared to GANs in different anatomical sites, such as the lung [15] and brain [16]. To further improve the fidelity of sCT, energy-guided diffusion models [17] and frequency-guided diffusion models [18,19] have attracted attention for their efficacy in both paired and unpaired settings. Despite these advances, diffusion models often struggle to reconstruct anatomical consistency [20,21]. This may restrict the clinical utility of generated sCT images, especially when precise delineation of organs is required [22]. To mitigate this limitation, recent studies have introduced anatomical priors into diffusion models by utilizing segmentation masks obtained from planning CTs, which are acquired before treatment [23,24]. Conditioned on this anatomical guidance, diffusion models are capable of recovering the anatomical structures, thus improving spatial alignment between sCT and ground-truth CT images. Nonetheless, these models commonly rely on single-scale segmentation masks, which may be insufficient to capture both global anatomical contours and local structural details. This challenge also arises in medical image segmentation tasks, where multiscale representations have been widely adopted to address this limitation. By integrating textural information across different spatial resolutions, the model learns to handle both global and local features [25,26,27]. For instance, the recent segmentation network designs a multi-scale attention module to extract both detailed and broad features [27].

Motivated by these findings, we propose a multiscale segmentation-guided diffusion framework that takes advantage of anatomical priors in the form of mask pyramids. Segmentation masks are first generated using a state-of-the-art segmentation model and then downsampled to construct a hierarchy of coarse-to-fine guidelines. This mask pyramid is integrated into the conditional diffusion model to provide structural information across multiple scales. Fine-scale masks emphasize detailed local features, while coarse-scale masks preserve broader global information. To complement the multiscale module, we introduce a scale-specific loss function, which computes the mean squared error (MSE) at each scale. This multiscale supervision enables the model to capture both local and global anatomical features, therefore improving the anatomical accuracy of sCT images and advancing the clinical usability of CBCT-to-CT synthesis.

## 2. Materials and Methods

### 2.1. Dataset Preparation

We used the brain dataset from the SynthRAD2023 challenge (Task 2), a multi-center benchmark dataset for evaluating synthetic CT (sCT) generation algorithms [28]. It consists of 180 patients with paired CBCT and CT images collected from multiple institutions, covering diverse anatomical sites and acquisition settings. All CBCT and CT images were pre-aligned using a standardized registration pipeline provided by the challenge organizers [28]. No additional deformable registration or intensity-based refinements were applied. For image preprocessing, both CBCT and CT images were clipped to the intensity range of [−1000,3000] Hounsfield units (HU) to suppress outliers, followed by intensity normalization to [−1,1] for stable model training. All images were resampled to an in-plane resolution of 1.0 × 1.0 mm^2^ in the axial view.

**Figure 1:**
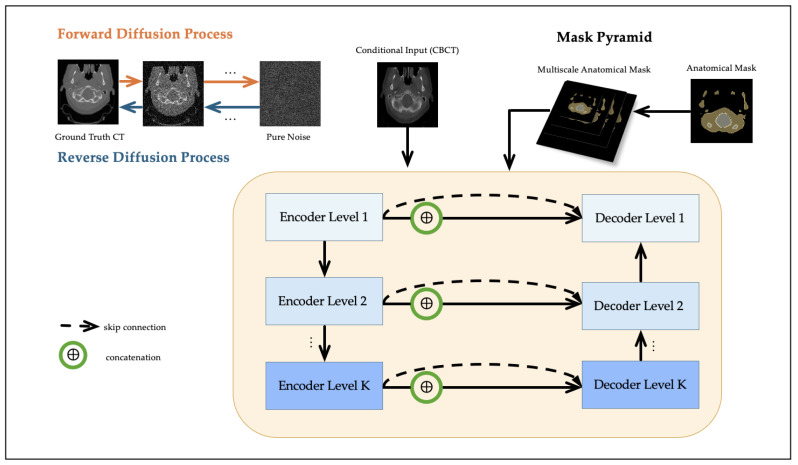
Workflow of the proposed multiscale segmentation-guided diffusion model. The diffusion model is conditioned on the CBCT image, and a segmentation mask pyramid is injected into the U-Net denoising network through feature concatenation at multiple scales.

Segmentation masks were generated from the CT scans using TotalSegmentator (https://github.com/wasserth/TotalSegmentator (accessed on 20 October 2025)) [29], which extracts 117 anatomical structures via a pre-trained nnU-Net [30]. These masks serve as anatomical priors and provide structural guidance to the diffusion model. Although the CT scans used for mask generation are also used as reference targets in training, the masks themselves are not employed as prediction labels. This setup reflects a clinically realistic scenario where anatomical priors, typically available from planning CTs, can assist in guiding CBCT-to-CT synthesis. While this approach does not exactly replicate the clinical use of planning CT-derived masks, it provides a practical approximation for training and evaluating segmentation-guided methods in the absence of dedicated planning CT data. The resulting masks were later used to construct the multiscale mask pyramid, as detailed in Section 2.3. An overview of the proposed framework is illustrated in Figure 1.

### 2.2. Conditional Diffusion Model

Our method is based on the conditional Denoising Diffusion Probabilistic Model (DDPM) [13], which generates synthetic CT (sCT) images $x_{0}$ from paired CBCT images *y* as conditioning inputs. The goal is to learn a conditional distribution $p_{\theta }\left(x_{0}|y\right)$ using a U-Net-based denoising network.

During the forward diffusion process, Gaussian noise is gradually added to the clean CT image $x_{0}$ over *T* steps:

(1) $$q\left(x_{t}|x_{0}\right)=N(x_{t};\;\sqrt{{\bar{\alpha }}_{t}}x_{0},\;\left(1-{\bar{\alpha }}_{t}\right)I),$$

where ${\bar{\alpha }}_{t}=\prod_{i=1}^{t}\left(1-\beta_{i}\right)$, and ${\left\{\beta_{t}\right\}}_{t=1}^{T}$ is a fixed variance schedule.

The reverse process denoises $x_{t}$ to estimate $x_{t-1}$ by predicting the added noise ϵ using a parameterized model $\epsilon_{\theta }\left(x_{t},t,y\right)$ conditioned on the CBCT input *y*. The reverse distribution is defined as:

(2) $$p_{\theta }\left(x_{t-1}|x_{t},y\right)=N(x_{t-1};\;\mu_{\theta }\left(x_{t},t,y\right),\;{\tilde{\beta }}_{t}I),$$

where the mean is computed as

(3) $$\mu_{\theta }\left(x_{t},t,y\right)=\frac{1}{\sqrt{\alpha_{t}}}\left(x_{t}-\frac{\beta_{t}}{\sqrt{1-{\bar{\alpha }}_{t}}}\epsilon_{\theta }\left(x_{t},t,y\right)\right),$$

and the posterior variance ${\tilde{\beta }}_{t}$ is

(4) $${\tilde{\beta }}_{t}=\frac{1-{\bar{\alpha }}_{t-1}}{1-{\bar{\alpha }}_{t}}\beta_{t}.$$

The training objective minimizes the MSE between the true noise and the predicted noise:

(5) $$L_{\mathrm{DDPM}}=E_{x_{0},\epsilon ,t}\left[∥\epsilon -\epsilon_{\theta }\left(x_{t},t,y\right){∥}^{2}\right].$$

In our implementation, the CBCT image *y* is concatenated with the noisy CT input $x_{t}$ along the channel dimension and jointly passed through the denoising U-Net at each timestep. This conditioning allows the network to leverage anatomical structures present in the CBCT to better reconstruct the clean CT scans.

### 2.3. Multiscale Mask Pyramid Module

While conditional diffusion models effectively restore intensity distributions, they often struggle to recover subtle soft-tissue structures due to the lack of anatomical guidance. To address this limitation, we propose a multiscale module based on a mask pyramid that encodes anatomical priors at multiple spatial resolutions.

A high-resolution anatomical segmentation mask $M_{0}$ is first generated for each patient using TotalSegmentator [29]. The binary mask $M_{0}$ is subsequently downsampled using nearest-neighbor interpolation to construct a set of multiscale masks, denoted as $\left\{M_{1},M_{2},\ldots ,M_{K}\right\}$, where each $M_{k}$ captures structural information at a distinct spatial scale *k*. Each level of the mask pyramid $M_{k}$ is spatially aligned with a corresponding decoder level in the UNet. Let $h_{k}$ denote the feature map at resolution level *k*. A lightweight convolutional embedding $f(M_{k})$ is applied to the segmentation mask, and the result is concatenated with $h_{k}$ to obtain the anatomically conditioned feature map $h_{k}^{\prime }$:

(6) $$h_{k}^{\prime }=\mathrm{Concat}\left(h_{k},\;f\left(M_{k}\right)\right).$$

This design enables hierarchical anatomical conditioning, where coarse-scale masks (e.g., $M_{3}$) support global structure alignment in deeper layers and fine-scale masks (e.g., $M_{0}$) guide local detail reconstruction in shallower layers. Unlike prior methods that rely on single-scale anatomical masks [23,24], our framework incorporates explicit multiscale anatomical guidance in both the network architecture and the loss function (described in Section 2.4). This allows the model to leverage anatomical priors across multiple receptive fields, which is particularly beneficial in anatomically heterogeneous regions such as bone–soft tissue boundaries.

### 2.4. Multiscale Anatomical Loss

To enhance anatomical supervision during training, we propose a multiscale anatomical loss that provides explicit guidance at each resolution level of the mask pyramid. At each scale *k* (for k=0,1,…,K), we compute the MSE between the predicted noise $\epsilon_{\theta }^{\left(k\right)}$ and the ground-truth noise $\epsilon^{\left(k\right)}$, restricted to the spatial region defined by the corresponding downsampled segmentation mask $M_{k}$. Formally, the total training objective is formulated as

(7) $$L_{\mathrm{total}}=L_{\mathrm{DDPM}}+\sum_{k=0}^{K}w_{k}\cdot E\left[{∥M_{k}⊙\left({\tilde{\epsilon }}_{\theta }^{\left(k\right)}-{\tilde{\epsilon }}^{\left(k\right)}\right)∥}^{2}\right],$$

where ⊙ denotes element-wise multiplication, and $w_{k}$ is the weight assigned to scale *k*.

This formulation enables the diffusion model to receive anatomically structured supervision across multiple spatial scales, allowing it to capture both coarse context and fine soft-tissue details during the denoising process. Further implementation details regarding the initialization and optimization of the scale weights $w_{k}$ are provided in Section 2.6.

### 2.5. Evaluation Metrics

We evaluated the accuracy of synthetic CT (sCT) generation using both standard image similarity metrics and clinically meaningful dose-relevant indicators. All metrics are computed slice-wise and then averaged across the test set. Let sCT(i,j) and CT(i,j) denote the predicted and ground-truth CT intensities at pixel (i,j), over an image of size $n_{x}\times n_{y}$.

Mean Absolute Error (MAE) measures the average voxel-wise intensity difference:
  (8) $$\mathrm{MAE}=\frac{1}{n_{x}n_{y}}\sum_{i,j}\left|\mathrm{sCT}(i,j)-\mathrm{CT}(i,j)\right|.$$
Peak Signal-to-Noise Ratio (PSNR) is a logarithmic metric quantifying signal fidelity:
  (9) $$\mathrm{PSNR}=10\times \log_{10}\left(\frac{{\mathrm{MAX}}^{2}}{\frac{1}{n_{x}n_{y}}\sum_{i,j}{\left|\mathrm{sCT}(i,j)-\mathrm{CT}(i,j)\right|}^{2}}\right),$$
  where MAX denotes the dynamic range of intensities. In our study, we set MAX=4000 HU, corresponding to the intensity clipping range of [−1000,3000] HU.
Structural Similarity Index (SSIM) evaluates structural agreement in local image patches:
  (10) $$\mathrm{SSIM}\left(x,y\right)=\frac{\left(2\mu_{x}\mu_{y}+C_{1}\right)\left(2\sigma_{xy}+C_{2}\right)}{\left(\mu_{x}^{2}+\mu_{y}^{2}+C_{1}\right)\left(\sigma_{x}^{2}+\sigma_{y}^{2}+C_{2}\right)},$$
  where *x* and *y* are local image patches from sCT and CT; μ, $\sigma^{2}$, and $\sigma_{xy}$ denote mean, variance, and covariance. Constants $C_{1}$ and $C_{2}$ are defined as $C_{1}={\left(0.01L\right)}^{2}$ and $C_{2}={\left(0.03L\right)}^{2}$, where *L* is the dynamic range of the image. We set L=4000, corresponding to the intensity clipping range.
HU Bias reflects the global intensity offset between sCT and reference CT, which is critical for dose calculation. A smaller HU bias (ideally close to 0) indicates better calibration of synthetic intensities:
  (11) $$\mathrm{HU}\;\mathrm{Bias}=\frac{1}{n_{x}n_{y}}\sum_{i,j}\left(\mathrm{sCT}(i,j)-\mathrm{CT}(i,j)\right).$$
Gradient Root Mean Square Error (Grad-RMSE) is a common metric to assess anatomical sharpness and structural fidelity:
  (12) $$\mathrm{Grad}-\mathrm{RMSE}=\sqrt{\frac{1}{n_{x}n_{y}}\sum_{i,j}{\left|\nabla \mathrm{sCT}(i,j)-\nabla \mathrm{CT}(i,j)\right|}^{2}}.$$
  where ∇ denotes the image gradient operator (e.g., Sobel or central difference), capturing spatial intensity changes. Lower Gradient RMSE indicates better preservation of anatomical boundaries, making it particularly important for downstream applications such as segmentation or dose calculation near organ interfaces.
Dice Similarity Coefficient (DSC) is computed to quantify the structural agreement between anatomical segmentations derived from sCT and CT images. Specifically, we apply tools such as TotalSegmentator to extract segmentation masks from both domains and compute their overlap:
  (13) $$\mathrm{DSC}=\frac{2|A\cap B|}{\left|A|+|B\right|},$$
  where *A* and *B* denote the predicted and reference segmentation masks. A higher Dice score indicates improved anatomical consistency and potentially greater dosimetric reliability.
To quantify tissue-wise accuracy, we also compute the MAE within three HU-defined anatomical regions: air (HU < −500), soft tissue (HU between −500 and 300), and bone (HU > 300). Let $\Omega_{r}$ denote the set of pixels belonging to region *r*. The region-specific MAE is computed as
  (14) $${\mathrm{MAE}}_{r}=\frac{1}{|\Omega_{r}|}\sum_{\left(i,j\right)\in \Omega_{r}}\left|\mathrm{sCT}(i,j)-\mathrm{CT}(i,j)\right|.$$
  These region-specific errors provide a more clinically meaningful evaluation of synthetic CT accuracy, particularly in capturing air-tissue interfaces, soft tissue contrast, and bone density.

### 2.6. Implementation Details

All experiments were conducted on NVIDIA A100 GPUs. The base architecture is a conditional DDPM with the UNet backbone comprising residual and attention blocks, following Peng et al. [16]. The UNet consists of four resolution levels with feature channel dimensions of [128,256,512,1024]. Each level contains two residual blocks, and attention layers are applied at the two lowest resolutions (i.e., spatial sizes of 64 and 32).

The diffusion process uses T=1000 timesteps with a cosine noise scheduler. We used the Adam optimizer with an initial learning rate of $1\times 10^{-4}$, cosine annealing learning rate decay, and a batch size of 4. All models were trained for 200 epochs using automatic mixed precision (AMP) to accelerate convergence. During training, we evaluated model performance on the validation set after each epoch. The checkpoint with the lowest validation loss was selected for final testing.

For anatomical supervision, a segmentation mask pyramid $\{M_{0},M_{1},M_{2}$$,M_{3}\}$ was constructed by downsampling the full-resolution binary mask $M_{0}$. Each scale-specific mask $M_{k}$ is spatially aligned with a decoder stage in the UNet, and its embedding is injected as described in Section 2.3. To assess the effect of multiscale anatomical guidance, we experimented with different numbers of active mask levels, e.g., K=0 (only including $M_{0}$), K=1 (using $\left\{M_{0},M_{1}\right\}$), and K=2 (using $\left\{M_{0},M_{1},M_{2}\right\}$). For each setting, the anatomical loss was only applied at the selected scales. The scale weights $w_{k}$ were initialized as $w_{k}=\left\{1.0,0.5,0.25,0.125\right\}$ and implemented as learnable scaler parameters jointly optimized with the model.

To prevent trivial minimization of the anatomical loss (e.g., all $w_{k}$ reduces to 0), we applied Dropout (p=0.5) in the decoder and used early stopping based on validation loss. These implicit regularization strategies encouraged the model to retain meaningful anatomical supervision at multiple scales. Empirically, all scale weights remained non-zero throughout training.

## 3. Results

### 3.1. Experimental Setup

We conducted all experiments on the SynthRAD2023 brain dataset, which was split at the patient level into 160 training, 20 validation, and 20 testing cases using a fixed random seed to ensure reproducibility. To prevent data leakage, each patient was assigned to only one subset. All CBCT and CT volumes were preprocessed by clipping to a fixed intensity range of [−1000,3000] HU and normalized to [−1,1]. During evaluation, predictions were rescaled to HU using the inverse of the normalization transformation.

All metrics were computed on 2D axial slices and then averaged across the entire test set. We reported MAE, PSNR, SSIM, HU bias, gradient RMSE, DSC, and region-specific MAE (air, soft tissue, bone). SSIM was calculated per slice using a Gaussian window and masked to exclude background voxels. As a reference baseline, we also included CBCT-versus-CT comparisons to reflect the initial image fidelity prior to synthesis.

### 3.2. Ablation Study

We compared the following configurations to systematically evaluate the impact of anatomical guidance and multiscale supervision.

- CBCT: Raw CBCT images directly compared with the ground-truth CT, without any synthesis.
- Baseline Diffusion [16]: A conditional DDPM with a UNet backbone, trained without anatomical supervision.
- SegGuided Diffusion (Single-scale, K=0) [23]: The baseline DDPM trained with a single binary anatomical mask derived from TotalSegmentator as structural guidance.
- Multiscale SegGuided Diffusion (K=1/2/3): An extension of the SegGuided model that incorporates a multiscale mask pyramid by downsampling the original mask to *K* additional resolutions, aligned with the encoder. Here, *K* denotes the number of auxiliary mask levels beyond the full-resolution input $M_{0}$.
- Multiscale SegGuided Diffusion with Multiscale Loss: The above model is further enhanced by a multiscale anatomical loss applied at each decoder resolution to enforce anatomical consistency across scales.

All models were trained under identical optimization settings and number of training iterations. To ensure fair comparison, the model architecture was kept constant across all ablation variants. The number of parameters (83.4M), FLOPs (479.1 GFLOPs), and inference time (around 30 s per 2D slice) remained consistent, as all anatomical branches and mask embeddings were pre-defined in the model and dynamically activated during inference.

Quantitative results on the testing set are summarized in Table 1. The raw CBCT input shows substantial deviation from ground-truth CT, with a high MAE of 507.32 HU and a low SSIM of 0.65, underscoring the need for accurate sCT synthesis in clinical practice. The baseline diffusion model significantly reduces this gap, achieving an MAE of 69.78 HU and an SSIM of 0.87, demonstrating the efficacy of conditional diffusion models for sCT generation. Introducing single-scale anatomical guidance (SegGuided Diffusion, K=0) further improves performance across all metrics, confirming the importance of structural supervision. Extending to a multiscale anatomical module yields the best performance at K=2, reaching an MAE of 62.69 HU and SSIM of 0.89. We observed slightly worse results at K=1 (possibly due to limited hierarchical context), and diminishing returns at K=3 (suggesting oversaturation of redundant spatial priors).

Ultimately, the combination of multiscale anatomical conditioning and multiscale supervision loss yields the best overall performance, achieving an MAE of 61.82 HU and a PSNR of 32.05 dB. Compared to the original CBCT, this represents an 88% reduction in intensity error and an improvement of over 10 dB in image quality. These results highlight the potential of multiscale anatomical guidance to improve pixel-level accuracy and structural consistency in sCT generation, thereby promoting CBCT-to-CT translation for radiotherapy applications.

### 3.3. Quantitative Results

We compared our proposed multiscale framework with several baseline architectures for CBCT-to-CT translation, including CycleGAN [31], UNet [32], UNet++ [33], and Swin-UNet [34], as summarized in Table 2.

Compared to the raw CBCT input, which exhibits poor image fidelity (MAE: 507.32 HU, SSIM: 0.65) and large HU bias (−219.36 HU), all deep learning-based models reduce reconstruction error. CycleGAN improves overall structure but still suffers from high variance and residual HU bias, possibly due to mode collapse or training instability. Swin-UNet, a transformer-based architecture, offers moderate improvements, but its performance is generally comparable to that of the GAN model. In contrast, UNet and UNet++ achieve lower MAEs (64.60 HU and 62.04 HU, respectively) and reduce HU bias to ±5 HU. They also improve region-specific accuracy, particularly in air and bone regions, and achieve DSC exceeding 0.94.

Our proposed model, Multiscale SegGuided Diffusion, further outperforms these baseline models across all metrics. Notably, the model combining multiscale anatomical priors with multiscale supervision (Multiscale SegGuided Diffusion with Multiscale Loss) achieves the best results: PSNR of 32.05 dB, SSIM of 0.90, and a nearly zero HU bias (−0.72 HU). It also yields the lowest gradient RMSE (696.86), indicating improved preservation of anatomical boundaries. For tissue-specific metrics, our model reports the lowest MAEs across air (21.0 HU), soft tissue (34.84 HU), and bone (113.06 HU), demonstrating its robustness across different regions.

**Figure 2:**
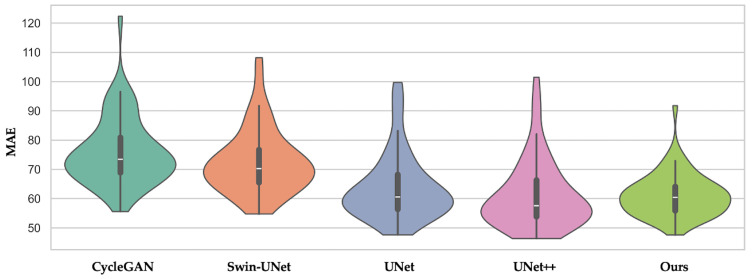
Violin plot of MAE across patients for CycleGAN, Swin-UNet, UNet, UNet++, and our proposed method. The distribution shows that our method not only achieves lower average MAE but also exhibits less inter-patient variance.

As shown in Figure 2, our method exhibits both the lowest average MAE and the narrowest interquartile range among all the compared methods. In contrast, CycleGAN and Swin-UNet display wider spreads with higher upper tails, indicating less consistent performance among patients. Combined with the results of Table 2, this supports the clinical robustness of our multiscale-guided framework.

Together, these findings suggest that integrating multiscale anatomical guidance and multiscale supervision into the diffusion framework not only enhances image quality but also improves clinical reliability by reducing HU error and and preserving anatomical consistency.

### 3.4. Qualitative Results

**Figure 3:**
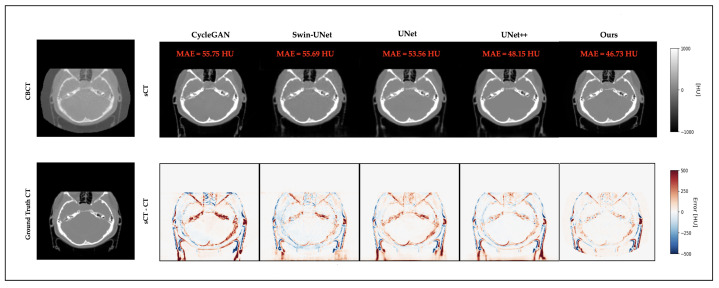
Qualitative comparison on a representative brain slice. Top: CBCT and sCT images generated by different models. Bottom: ground-truth CT and error maps compared to ground-truth CT.

**Figure 4:**
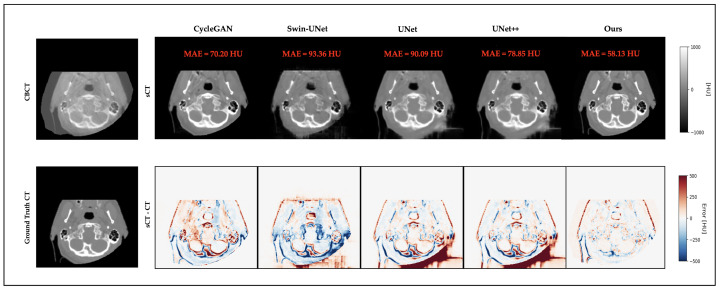
Another representative example of a brain slice. Top: CBCT and sCT images generated by different models. Bottom: ground-truth CT and error maps compared to ground-truth CT.

Figure 3 and Figure 4 present representative cases from the testing set. Both include sCT images predicted by different models, as well as the corresponding error maps compared to the ground-truth CT scans. As seen in both cases, UNet++ successfully recovers anatomical outlines but tends to produce blurring in soft-tissue regions. In contrast, our proposed Multiscale SegGuided Diffusion model supervised with multiscale loss displays improved anatomical contrast and edge continuity. These qualitative observations are consistent with the quantitative results reported in Table 1, denoting the significance of multiscale anatomical guidance to improve both numerical precision and visual quality.

**Figure 5:**
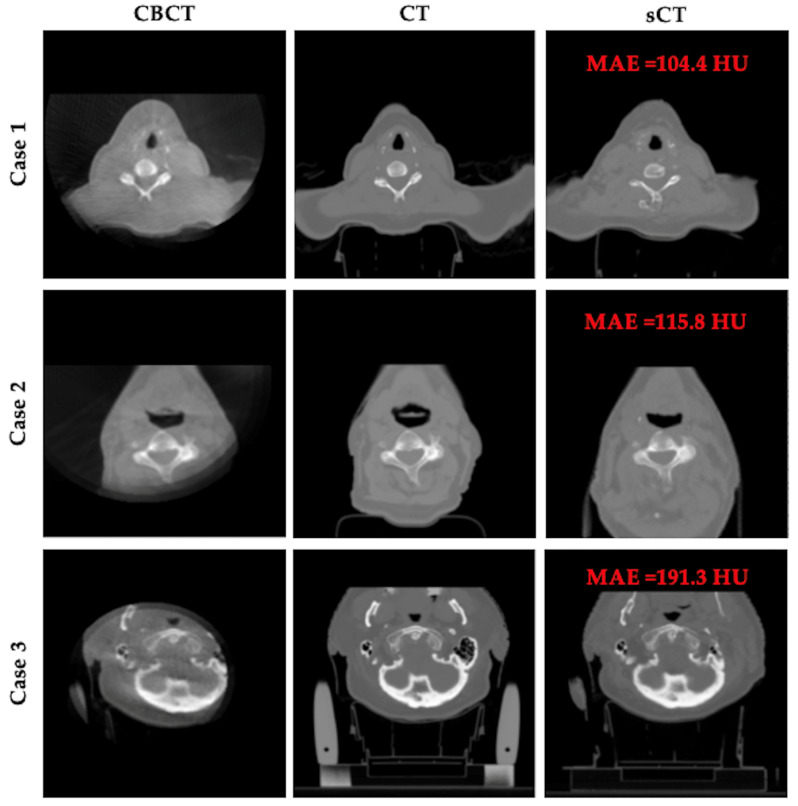
Representative failure cases. Each row shows CBCT, planning CT, and sCT results for a patient.

Despite the overall strong performance of our model, Figure 5 presents three representative failure cases. In these examples, the presence of severe artifacts and limited field of view (FOV) in the CBCT inputs significantly hinders model performance. These challenges result in missing tissues or anatomical distortions in the generated sCT images, revealing the limitations of our model under incomplete or noisy acquisition conditions.

## 4. Discussion

This study aims to evaluate whether incorporating multiscale segmentation guidance into a diffusion framework can enhance the fidelity and anatomical consistency of sCT images generated from CBCT inputs. While diffusion models have demonstrated remarkable success in medical image synthesis due to stability and generative capacity [15,16,35,36], their lack of explicit anatomical guidance can result in suboptimal structural accuracy, especially in critical soft-tissue regions [23,24,37].

To address this limitation, segmentation-based anatomical priors were introduced into the diffusion process, where they demonstrated their efficacy in significantly enhancing structural consistency [23,24]. Building upon this direction, we propose a multiscale segmentation-guided framework, motivated by the hypothesis that anatomical priors should not be limited to a single spatial scale. The multiscale module has been proven to improve model performance in medical image segmentation by capturing both local anatomical details and global structural information [25,26,27]. To synchronize the multiscale anatomical guidance, we introduce a multiscale loss function that supervises structural consistency at each resolution level. Both quantitative and qualitative results demonstrate that our proposed framework not only decreases intensity error between original CBCT and reference CT images but also reconstructs better anatomical details.

Several recent works have explored the SynthRAD2023 Task 2 dataset, offering diverse solutions to the CBCT-to-CT translation problem. In ARTInp [38], a two-stage framework is proposed that first inpaints missing CBCT regions before applying a translation model, achieving a PSNR of around 27 dB. STF-RUE [24] combined single-mask segmentation guidance with uncertainty quantification, highlighting the clinical relevance of structure-aware synthesis. GLFC [39] introduced Mamba-enhanced global–local contrast learning and achieved a top SSIM of 0.915. These methods underscore the importance of anatomical fidelity and model robustness. Compared to these approaches, our proposed Multiscale SegGuided Diffusion model achieves competitive or superior performance, with a PSNR of 32.05 dB and SSIM of 0.90 while maintaining nearly zero HU bias. In contrast to ARTInp, which relies on explicit image inpainting, our framework operates end-to-end and dynamically incorporates multiscale anatomical priors during denoising, with an additional multiscale supervision loss at multiple resolutions. Although STF-RUE focuses on uncertainty bounds, our method emphasizes structural guidance through multiscale supervision, although it faces challenges in regions with severe CBCT artifacts or truncated fields of view. Taken together, our results demonstrate that diffusion models incorporated with multiscale segmentation priors offer a promising direction for robust and anatomically faithful CBCT-to-CT translation.

However, our approach has several limitations. First, the current framework requires both segmentation masks and CBCT images during training, which may not be readily available in various clinical settings. Second, while our method improves the overall quality of sCT images, it struggles to restore fine anatomical details in high-frequency regions such as the skull. To address this, future work will investigate the integration of frequency-guided diffusion models [18,19] and anatomical priors. Additionally, the current implementation is based on 2D slices, which may restrict spatial continuity and volumetric consistency. Extending the framework to a 3D model can further strengthen its clinical usability in sCT generation. To assess the generalizability of the model, we will also incorporate center-held-out validation schemes where all patients from one institution are excluded from training. This will allow a more rigorous evaluation of domain robustness under institutional shifts. Future work also includes evaluating dose-aware endpoints such as DVH and gamma analysis once treatment plans become available.

## 5. Conclusions

In this study, we proposed a multiscale segmentation-guided diffusion framework for CBCT-to-CT synthesis. By integrating a multiscale segmentation mask pyramid and a scale-specific anatomical loss, our method demonstrates improved fidelity and anatomical consistency in synthetic CT images on the SynthRAD2023 brain dataset. While the results show promising improvements over baseline diffusion and convolutional models, they are currently validated on a single anatomical site. Future extensions will involve 3D volumetric models, multi-site validation, and assessments toward real-time clinical deployment in adaptive workflows.

## Figures and Tables

**Table 1:** Ablation study evaluating the impact of anatomical supervision and multiscale components on CBCT-to-CT synthesis. ↓ stands for lower is better, and ↑ stands for higher is better. The bold values indicate the best performance across all methods for each metric.

Method	MAE (↓)	PSNR (↑)	SSIM (↑)
CBCT	507.32 ± 470.80	20.48 ± 7.49	0.65 ± 0.20
Baseline Diffusion [16]	69.78 ± 46.09	30.63 ± 3.47	0.87 ± 0.09
SegGuided Diffusion (K=0) [23]	64.15 ± 34.86	30.90 ± 3.32	0.88 ± 0.07
Multiscale SegGuided Diffusion (K=1)	65.06 ± 35.93	29.47 ± 4.05	0.88 ± 0.08
Multiscale SegGuided Diffusion (K=3)	63.1 ± 36.61	30.87 ± 4.15	0.88 ± 0.08
Multiscale SegGuided Diffusion (K=2)	62.69 ± 32.83	31.83 ± 3.34	0.89 ± 0.07
Multiscale SegGuided Diffusion with Multiscale Loss (K=2)	**61.82 ± 30.59**	**32.05 ± 3.27**	**0.90 ± 0.05**

**Table 2:** Performance of different architectures for CBCT-to-CT translation. ↓ stands for lower is better, and ↑ stands for higher is better. The bold values indicate the best performance across all methods for each metric.

Method	MAE (↓)	PSNR (↑)	SSIM (↑)	HU Bias (↓)	Grad-RMSE (↓)	DSC (↑)	MAE_air_ (↓)	MAE_soft_ (↓)	MAE_bone_ (↓)
CBCT	507.32 ± 470.80	20.48 ± 7.49	0.65 ± 0.20	−219.36 ± 141.52	2631.38 ± 363.70	0.0001 ± 0.0003	962.54 ± 12.75	56.90 ± 3.01	852.62 ± 112.24
CycleGAN	75.61 ± 77.43	29.99 ± 3.80	0.87 ± 0.10	−7.82 ± 9.77	1092.85 ± 171.46	0.84 ± 0.04	29.75 ± 12.68	61.09 ± 27.58	191.24 ± 86.50
Swin-UNet	73.13 ± 44.16	30.49 ± 3.21	0.87 ± 0.85	−5.21 ± 10.09	980.44 ± 160.21	0.86 ± 0.03	32.13 ± 13.13	52.5 ± 19.41	165.83 ± 74.65
UNet	64.60 ± 40.07	31.18 ± 3.37	0.89 ± 0.78	3.42 ± 8.21	765.14 ± 295.74	0.94 ± 0.02	24.15 ± 12.71	41.83 ± 16.95	127.9 ± 64.12
UNet++	62.04 ± 40.35	31.25 ± 3.52	0.89 ± 0.79	4.1 ± 7.9	730.56 ± 310.23	0.94 ± 0.02	23.1 ± 12.36	40.56 ± 15.93	124.65 ± 63.38
Multiscale SegGuided Diffusion + Multiscale Loss (Ours)	**61.82 ± 30.59**	**32.05 ± 3.27**	**0.90 ± 0.05**	**−0.72 ± 7.13**	**696.86 ± 341.59**	**0.95 ± 0.02**	**21.0 ± 12.49**	**34.84 ± 14.58**	**113.06 ± 62.07**

## Data Availability

The original data presented in the study are openly available in Zenodo at https://doi.org/10.5281/zenodo.7260704 or [28].

## Abbreviations

The following abbreviations are used in this manuscript:

CT	Computed tomography
sCT	Synthetic CT
CBCT	Cone-beam computed tomography
IGART	Image-guided adaptive radiotherapy
GAN	Generative adversarial networks
HU	Hounsfield units
DDPM	Denoising diffusion probabilistic model
MSE	Mean squared error
MAE	Mean absolute error
PSNR	Peak signal-to-noise ratio
SSIM	Structural similarity index
Grad-RMSE	Gradient mean squared error
DSC	Dice similarity coefficient
